# Supplementary material for: Studying relative RNA localization from nucleus to the cytosol
Source: NAR Genom Bioinform. 2025 Jun 20;7(2):lqaf032. doi: 10.1093/nargab/lqaf032 (PMC12204760; doi:10.1093/nargab/lqaf032)
Supplement: lqaf032_Supplemental_File [file lqaf032_supplemental_file.pdf]

Studying relative RNA localization from nucleus to the cytosol

Supplementary Data

Supplementary Figures

Supplementary Figure S1.	Simulated data construction schema.	2
Supplementary Figure S2.	Comparison of β estimates.	3
Supplementary Figure S3.	Comparison of β estimates in subsampled data.	4
Supplementary Figure S4.	Proportion of consistently localized transcripts (low - high expression).	5
Supplementary Figure S5.	Proportion of consistently localized transcripts (protein coding - long non coding).	6
Supplementary Figure S6.	LI distribution per transcript biotype across cell lines.	7
Supplementary Figure S7.	LI distribution of retained–intron transcripts across cell lines.	8

Supplementary Tables

Supplementary Table S1.	Single-cell data accession numbers.	9
Supplementary Table S2.	ENCODE data accession numbers.	10
Supplementary Table S3.	Simulated Data – β estimates	11
Supplementary Table S4.	ENCODE Data – β estimates	11
Supplementary Table S5.	Gene and transcript localization summary.	12

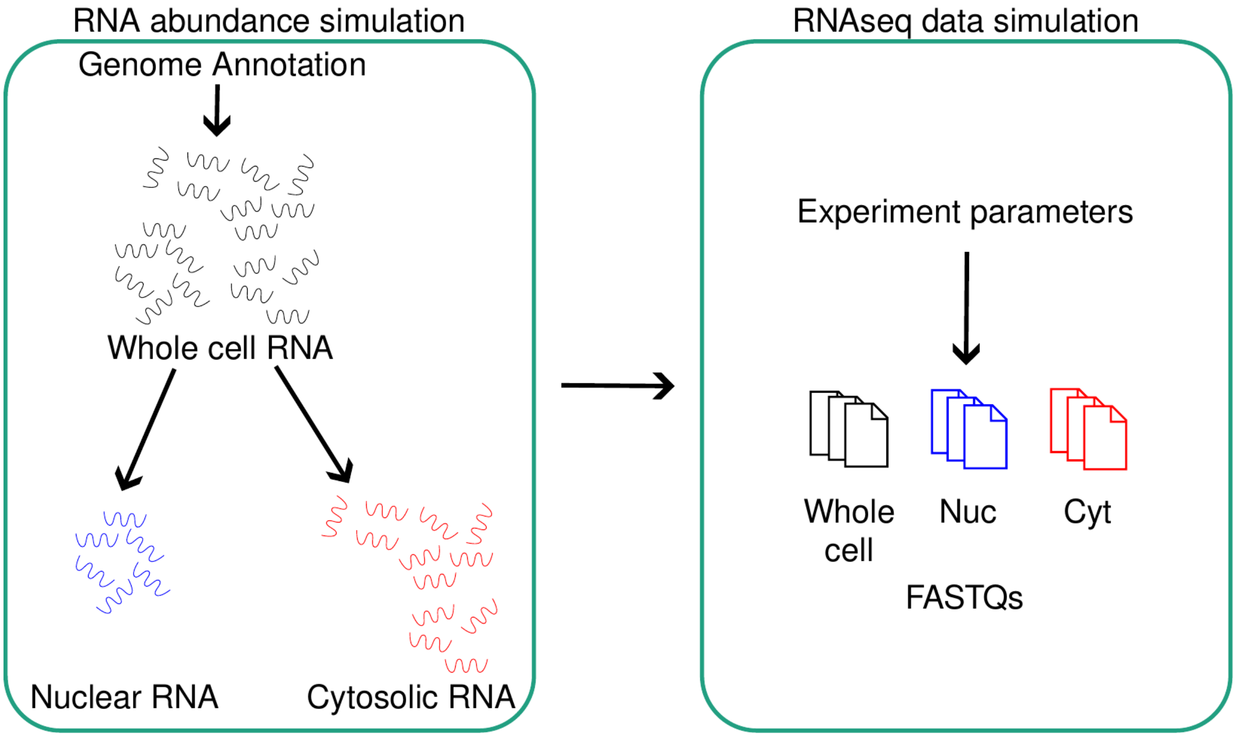

Figure S1. Simulated data construction schema. Description of the workflow followed to construct the simulated dataset. First, RNA abundance (number of molecules expressed per annotated transcript) for whole cell was simulated. This RNA abundance was split for every transcript into to two parts, one for the nuclear fraction and another one for the cytosolic fraction. This was repeated multiple times in order to produce simulated samples with diverse nucleo-cytosolic distribution. Finally, the simulated bulk RNAseq data was generated using the RNA abundances for whole cell, nuclear, and cytosolic fractions as starting points.

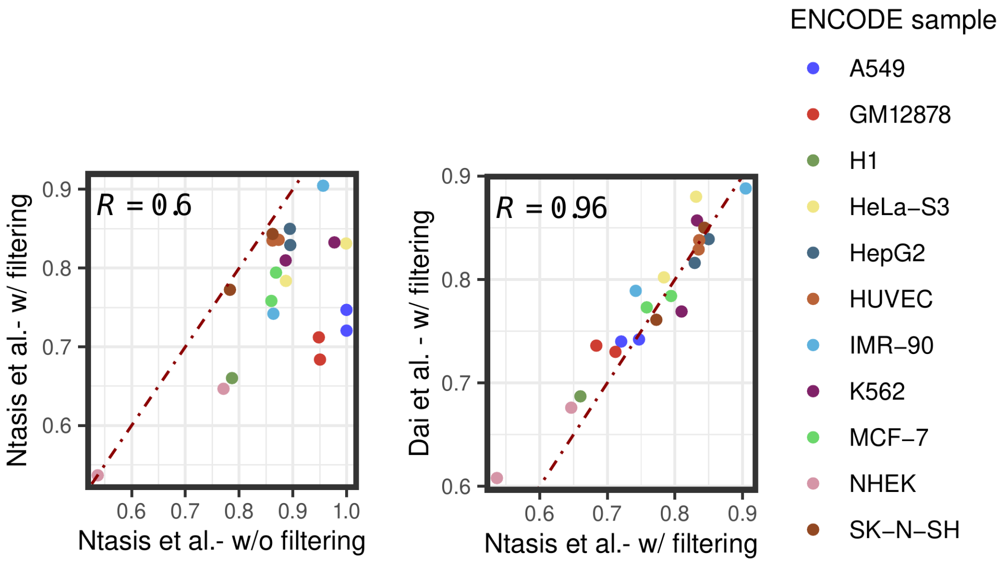

Figure S2. Comparison of β estimates depicted with scatter plots. The estimates were generated either based on the method proposed here or that presented by Dai et al. (1). Filtering is referring to the process of discarding transcripts with an outlier profile as described in Dai et al. (1). The dashed line represents the $y = x$ line. We also report the Pearson correlation coefficient for each pair of estimates illustrated in a scatter plot.

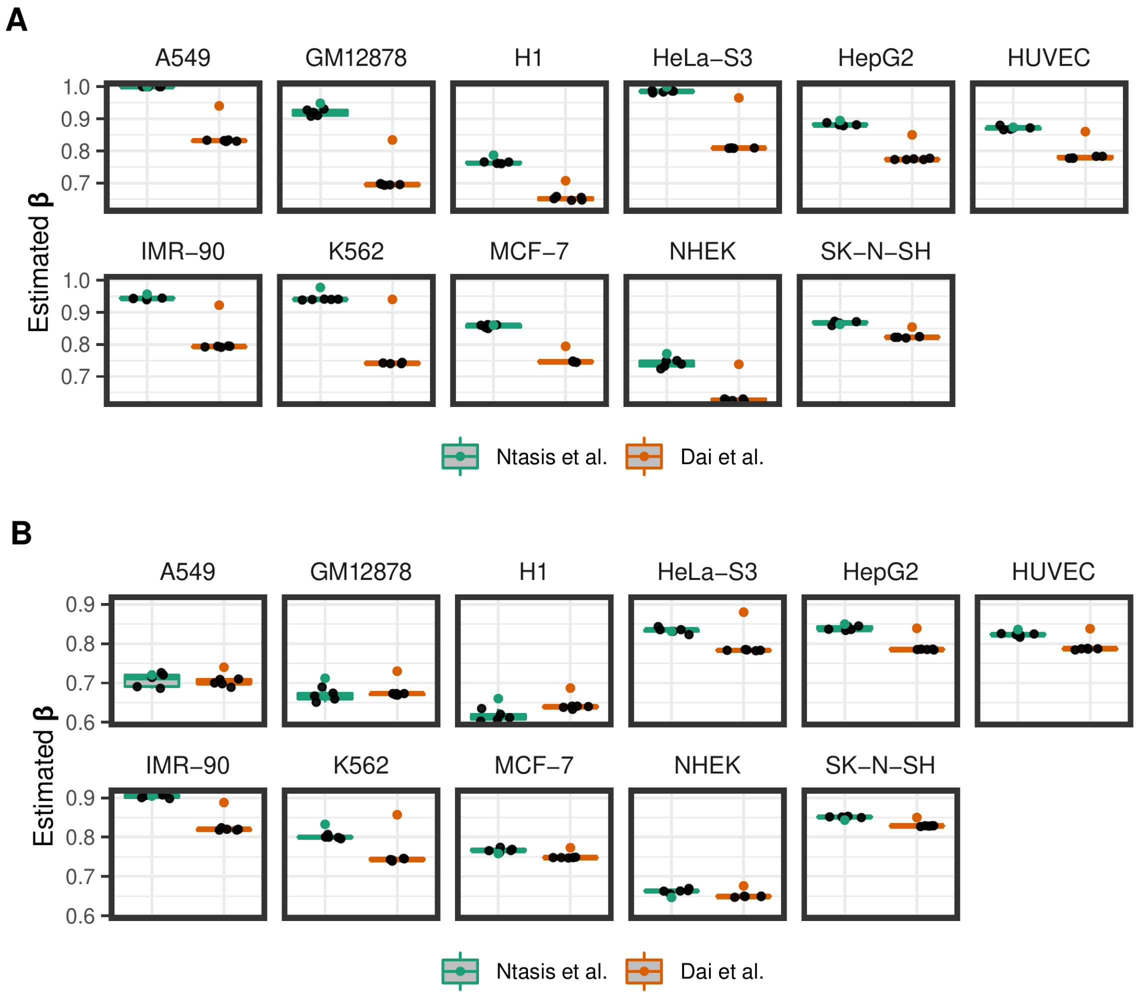

Figure S3. Comparison of β estimates in subsampled data. The estimates were generated either based on the method proposed here or that presented by Dai et al. (1). (A) Boxplots illustrating the distribution of β estimates computed from the subsampled data. The colored dots represent the original β estimate computed based on the whole dataset. The filtering approach of discarding transcripts with an outlier profile as described in Dai et al. (1) wasn't applied. (B) β estimates computed from the subsampled data after applying the aforementioned filtering.

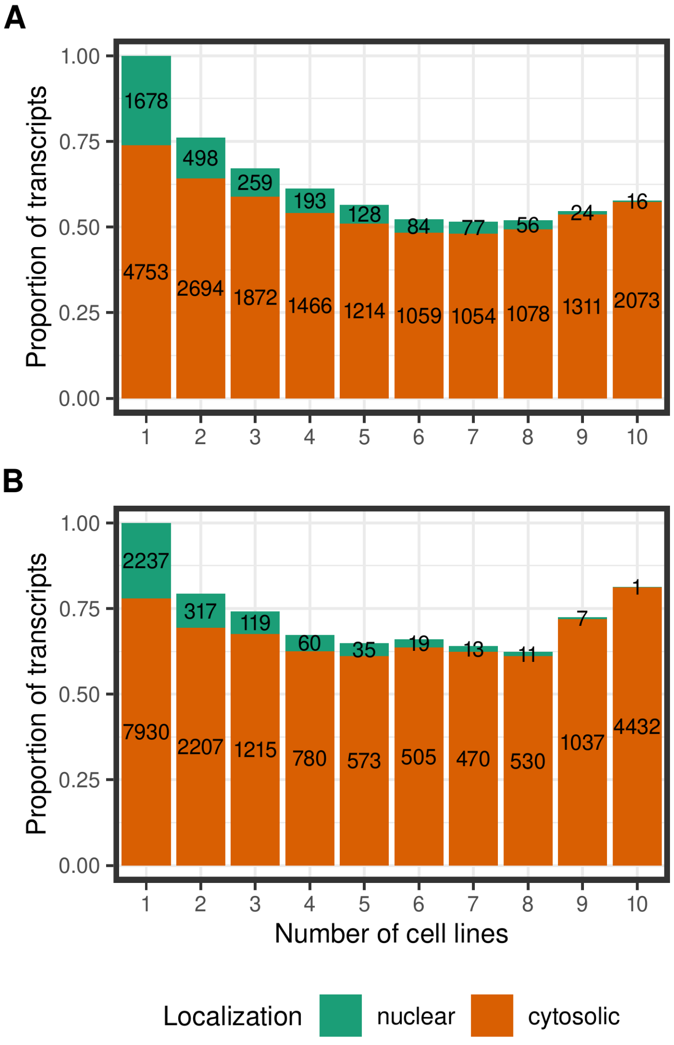

Figure S4. Proportion of consistently localized transcripts (low - high expression). Each barplot depicts the proportion of the transcripts, either lowly expressed (A), or highly expressed (B), that have the same localization, nuclear (localization index $(LI) \leq 0.5$) or cytosolic $(LI \geq 0.5)$, across all the cell lines in which they were found to be expressed. The number of transcripts each proportion corresponds to is also indicated. The x-axis displays the number of cell lines that the corresponding number of transcripts were detected. A cutoff of 2 FPKM in at least one cell line was considered (in the whole-cell samples) in order to separate lowly from highly expressed transcripts.

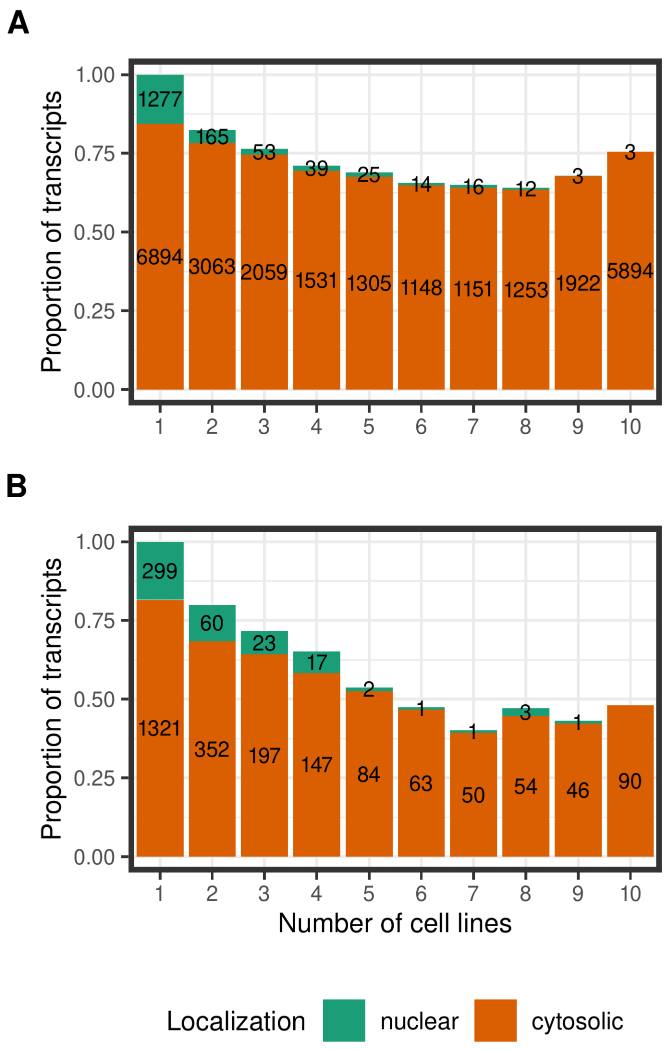

Figure S5. Proportion of consistently localized transcripts (protein coding - long non coding). Each barplot depicts the proportion of the transcripts, either protein coding (A), or long non coding (B), that have the same localization, nuclear (localization index $(LI) \leq 0.5$) or cytosolic $(LI \geq 0.5)$, across all the cell lines in which they were found to be expressed. The number of transcripts each proportion corresponds to is also indicated. The x-axis displays the number of cell lines that the corresponding number of transcripts were detected.

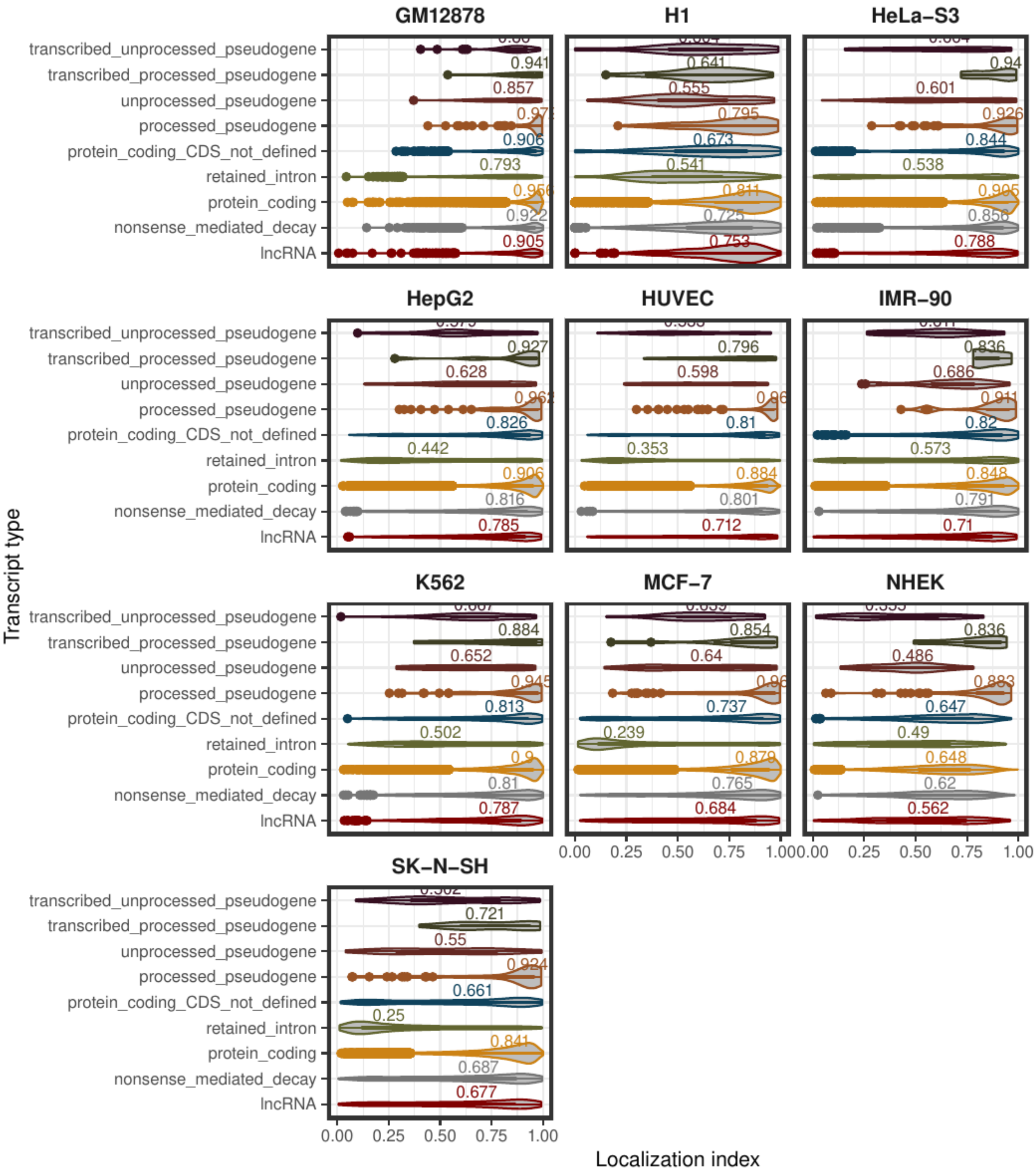

Figure S6. Localization index (LI) distribution per transcript biotype across cell line. The violin plots illustrate the LI distribution of transcripts annotated with a particular biotype, which are expressed in a specific cell line. The median LI value is indicated for every transcript biotype.

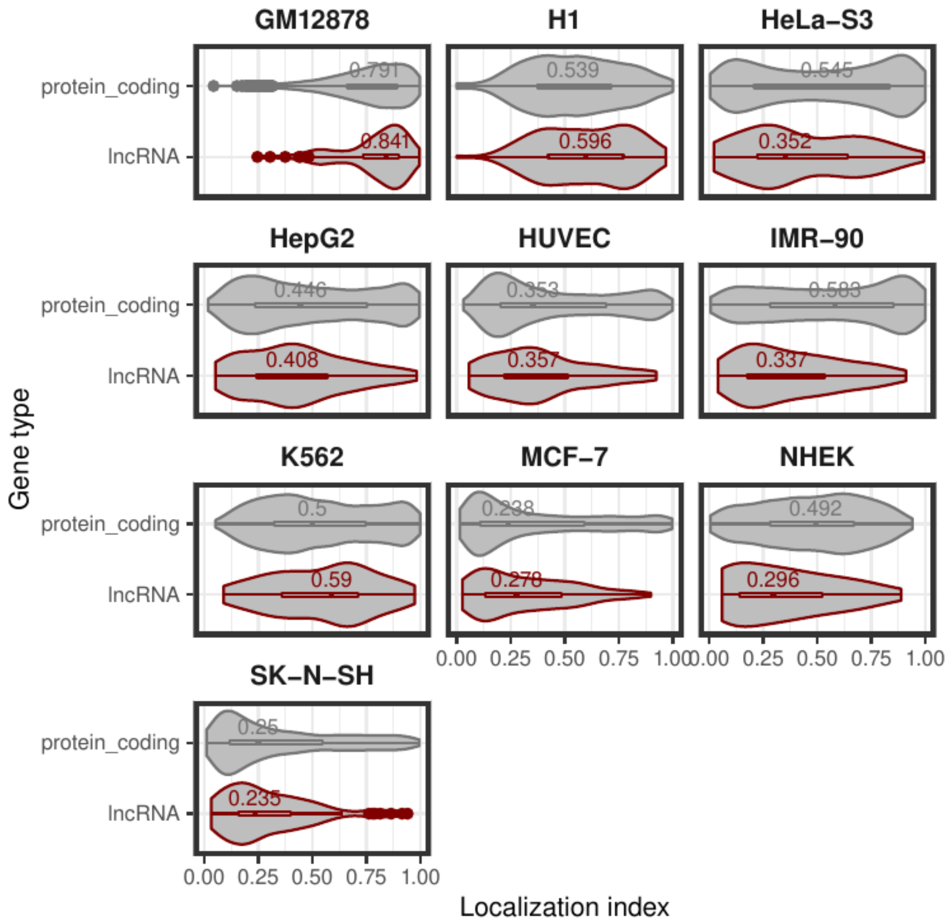

Figure S7. Localization index (LI) distribution of retained–intron transcripts across cell lines. The violin plots show for every cell line the distribution of LI of transcripts annotated as “retained intron”, and belong either to protein coding genes or to a lncRNA genes. The median LI value is indicated on top of the violin plots.

Run	Experiment	Sample	LibraryName
SRR6161494	SRX3273065	SRS2584457	C01
SRR6161495	SRX3273064	SRS2584457	C02
SRR6161496	SRX3273063	SRS2584457	C03
SRR6161497	SRX3273062	SRS2584457	C04
SRR6161490	SRX3273069	SRS2584457	C05
SRR6161491	SRX3273068	SRS2584457	C06
SRR6161492	SRX3273067	SRS2584457	C07
SRR6161493	SRX3273066	SRS2584457	C08
SRR6161499	SRX3273060	SRS2584457	C09
SRR6161500	SRX3273059	SRS2584457	C10
SRR6161529	SRX3273030	SRS2584457	C11
SRR6161530	SRX3273029	SRS2584457	C12
SRR6161539	SRX3273020	SRS2584457	N01
SRR6161587	SRX3272972	SRS2584457	N02
SRR6161458	SRX3273101	SRS2584457	N03
SRR6161457	SRX3273102	SRS2584457	N04
SRR6161477	SRX3273082	SRS2584457	N05
SRR6161478	SRX3273081	SRS2584457	N06
SRR6161575	SRX3272984	SRS2584457	N07
SRR6161481	SRX3273078	SRS2584457	N08
SRR6161564	SRX3272995	SRS2584457	N09
SRR6161608	SRX3272951	SRS2584457	N10
SRR6161498	SRX3273061	SRS2584457	N11
SRR6161501	SRX3273058	SRS2584457	N12
SRR6161524	SRX3273035	SRS2584457	S01
SRR6161523	SRX3273036	SRS2584457	S02
SRR6161522	SRX3273037	SRS2584457	S03
SRR6161521	SRX3273038	SRS2584457	S04
SRR6161520	SRX3273039	SRS2584457	S05
SRR6161519	SRX3273040	SRS2584457	S06
SRR6161502	SRX3273057	SRS2584457	S07
SRR6161503	SRX3273056	SRS2584457	S08
SRR6161504	SRX3273055	SRS2584457	S09
SRR6161505	SRX3273054	SRS2584457	S10
SRR6161506	SRX3273053	SRS2584457	S11
SRR6161507	SRX3273052	SRS2584457	S12

Table S1. Single-cell data accession numbers. Table with the Sequencing Read Archive (SRA) identifiers of the single-cell data used. We analyzed RNAseq data from total RNA (whole-cell) for 12 cells (S01-S12), and RNAseq from nuclear/cytosolic RNA for 12 cells as well (N01-N12, C01-C12).

quantification_file	bam_file	experiment	biosample	replicate	fraction
ENCFF590YJL	ENCFF773HDO	ENCSR000COU	H1	2	whole_cell
ENCFF254AJX	ENCFF950KXS	ENCSR000CPH	K562	1	whole_cell
ENCFF142FDI	ENCFF828HER	ENCSR000CPH	K562	2	whole_cell
ENCFF088ZTD	ENCFF218MGF	ENCSR000CTT	SK−N−SH	4	whole_cell
ENCFF457LLG	ENCFF666OAW	ENCSR000CTT	SK−N−SH	3	whole_cell
ENCFF081TVM	ENCFF687OMX	ENCSR000CON	A549	1	whole_cell
ENCFF329YJQ	ENCFF336EJL	ENCSR000CON	A549	2	whole_cell
ENCFF855XNI	ENCFF217OWU	ENCSR000CTQ	IMR−90	2	whole_cell
ENCFF742TTS	ENCFF768XGT	ENCSR000CTQ	IMR−90	1	whole_cell
ENCFF461CEJ	ENCFF311RKY	ENCSR000COQ	GM12878	1	whole_cell
ENCFF150FKV	ENCFF712TUU	ENCSR000COQ	GM12878	2	whole_cell
ENCFF268QJZ	ENCFF239GYE	ENCSR000CPL	NHEK	2	whole_cell
ENCFF085RUN	ENCFF994TIH	ENCSR000CPL	NHEK	1	whole_cell
ENCFF364YCB	ENCFF177JCJ	ENCSR000CPR	HeLa−S3	1	whole_cell
ENCFF008ARC	ENCFF090XWO	ENCSR000CPR	HeLa−S3	2	whole_cell
ENCFF030IUO	ENCFF895CTY	ENCSR000CPT	MCF−7	2	whole_cell
ENCFF296ZWB	ENCFF159NNM	ENCSR000CPT	MCF−7	1	whole_cell
ENCFF967OAC	ENCFF675NVY	ENCSR000COZ	HUVEC	1	whole_cell
ENCFF921ZWG	ENCFF456DGJ	ENCSR000COZ	HUVEC	2	whole_cell
ENCFF416RHJ	ENCFF947VHW	ENCSR000CPE	HepG2	1	whole_cell
ENCFF938LTW	ENCFF303VDN	ENCSR000CPE	HepG2	2	whole_cell
ENCFF978TLD	ENCFF850LBX	ENCSR000COK	K562	1	cytosolic
ENCFF505OOJ	ENCFF317WQG	ENCSR000COK	K562	2	cytosolic
ENCFF271NBH	ENCFF074PZH	ENCSR000COW	H1	2	nuclear
ENCFF979TFY	ENCFF523SST	ENCSR000CPB	HUVEC	3	nuclear
ENCFF176EST	ENCFF401JPO	ENCSR000CPB	HUVEC	4	nuclear
ENCFF139IKH	ENCFF500PZA	ENCSR000CTU	MCF−7	4	cytosolic
ENCFF999HBM	ENCFF844QFX	ENCSR000CTU	MCF−7	3	cytosolic
ENCFF360QYU	ENCFF154VSY	ENCSR000CTL	A549	4	cytosolic
ENCFF098TQL	ENCFF964FBY	ENCSR000CTL	A549	3	cytosolic
ENCFF602JOV	ENCFF118WJD	ENCSR000CPP	HeLa−S3	1	cytosolic
ENCFF798GHN	ENCFF132QVZ	ENCSR000CPP	HeLa−S3	2	cytosolic
ENCFF837TER	ENCFF546BFB	ENCSR000CPA	HUVEC	3	cytosolic
ENCFF188LTD	ENCFF788ZKW	ENCSR000CPA	HUVEC	4	cytosolic
ENCFF779WJW	ENCFF035JHQ	ENCSR000CPJ	NHEK	4	nuclear
ENCFF006SQI	ENCFF119KHR	ENCSR000CPJ	NHEK	3	nuclear
ENCFF104IQF	ENCFF560TMJ	ENCSR000CTN	IMR−90	1	cytosolic
ENCFF593JFS	ENCFF152VFL	ENCSR000CTN	IMR−90	2	cytosolic
ENCFF686EDQ	ENCFF009MEF	ENCSR000CTM	A549	3	nuclear
ENCFF632ALD	ENCFF926FOX	ENCSR000CTM	A549	4	nuclear
ENCFF301KCL	ENCFF927NZL	ENCSR000CPF	HepG2	1	cytosolic
ENCFF617CLP	ENCFF830RNQ	ENCSR000CPF	HepG2	2	cytosolic
ENCFF981MIW	ENCFF778RKA	ENCSR000CTR	SK−N−SH	4	cytosolic
ENCFF848UQF	ENCFF398LLD	ENCSR000CTR	SK−N−SH	3	cytosolic
ENCFF295JCI	ENCFF975HWR	ENCSR000CTP	IMR−90	2	nuclear
ENCFF698PFJ	ENCFF763NIZ	ENCSR000CTP	IMR−90	1	nuclear
ENCFF009WFT	ENCFF327DXV	ENCSR000CPS	K562	1	nuclear
ENCFF873PHU	ENCFF587FFX	ENCSR000CPS	K562	2	nuclear
ENCFF328QYM	ENCFF715DGD	ENCSR000CPK	NHEK	3	cytosolic
ENCFF749RLI	ENCFF576EEK	ENCSR000CPK	NHEK	4	cytosolic
ENCFF033JVM	ENCFF364IXG	ENCSR000CTO	MCF−7	4	nuclear
ENCFF786KJB	ENCFF125YQH	ENCSR000CTO	MCF−7	3	nuclear
ENCFF262EJQ	ENCFF181XMI	ENCSR000CPQ	HeLa−S3	1	nuclear
ENCFF622TLZ	ENCFF625ZJI	ENCSR000CPQ	HeLa−S3	2	nuclear
ENCFF613EYA	ENCFF762YBF	ENCSR000COR	GM12878	1	cytosolic
ENCFF862MNY	ENCFF630TBY	ENCSR000COR	GM12878	2	cytosolic
ENCFF700JEL	ENCFF128HEW	ENCSR000COV	H1	2	cytosolic
ENCFF192ZUM	ENCFF281BBM	ENCSR000CPC	HepG2	1	nuclear
ENCFF202BOX	ENCFF487IUM	ENCSR000CPC	HepG2	2	nuclear
ENCFF812UDN	ENCFF666MJD	ENCSR000CPO	GM12878	2	nuclear
ENCFF063DIU	ENCFF993NSG	ENCSR000CPO	GM12878	1	nuclear
ENCFF748ALF	ENCFF123ISS	ENCSR000CTS	SK−N−SH	4	nuclear
ENCFF216JTT	ENCFF437DAI	ENCSR000CTS	SK−N−SH	3	nuclear

Table S2. ENCODE data accession numbers. Table with the identifiers of the data used from the ENCODE project. For every biosample we analyzed in our study, we indicate the accession number of the respective experiment, transcript quantification file, and bam file used.

Simulated β	Ntasis et al.	Dai et al.
0.50	0.50	0.52
0.60	0.59	0.56
0.70	0.70	0.64
0.80	0.80	0.73

Table S3. Simulated Data – β estimates. Table with β estimates calculated from the simulated data using both our method and the one presented by Dai et al. (1). The true simulated value of β is indicated as well.

Sample	Biosample	Ntasis	Ntasis_filtered	Dai	Dai_filtered
A549_1	A549	1.00	0.72	0.94	0.74
A549_2	A549	1.00	0.75	0.87	0.74
GM12878_2	GM12878	0.95	0.68	0.85	0.74
GM12878_1	GM12878	0.95	0.71	0.83	0.73
HeLa-S3_1	HeLa-S3	1.00	0.83	0.96	0.88
HeLa-S3_2	HeLa-S3	0.89	0.78	0.81	0.80
K562_1	K562	0.98	0.83	0.94	0.86
K562_2	K562	0.89	0.81	0.77	0.77
IMR-90_1	IMR-90	0.96	0.91	0.92	0.89
IMR-90_2	IMR-90	0.86	0.74	0.76	0.79
HepG2_2	HepG2	0.90	0.83	0.83	0.82
HepG2_1	HepG2	0.89	0.85	0.85	0.84
endothelial_1	endothelial	0.87	0.84	0.86	0.84
endothelial_2	endothelial	0.86	0.83	0.84	0.83
MCF-7_2	MCF-7	0.87	0.79	0.81	0.78
MCF-7_1	MCF-7	0.86	0.76	0.79	0.77
SK-N-SH_3	SK-N-SH	0.86	0.84	0.85	0.85
SK-N-SH_4	SK-N-SH	0.78	0.77	0.76	0.76
H1_2	H1	0.79	0.66	0.71	0.69
keratinocyte_1	keratinocyte	0.77	0.65	0.74	0.68
keratinocyte_2	keratinocyte	0.54	0.54	0.62	0.61

Table S4. ENCODE Data – β estimates. Table with β estimates calculated from the ENCODE data using both our method and the one presented by Dai et al. (1). The two methods have been applied with and without the outlier filtering process described in Dai et al. (1).

measurement	GM12878	H1	HeLa−S3	HepG2	HUVEC	IMR−90	K562	MCF−7	NHEK	SK−N−SH
# genes expressed	11,611	14,289	11,565	11,818	12,008	10,866	11,230	12,433	11,621	12,732
# transcripts expressed	26,552	37,029	23,845	25,811	27,584	20,039	25,582	28,011	22,707	30,085
# cytosolic transcripts	25,268	27,517	20,553	20,855	21,967	16,084	20,611	21,353	13,073	21,339
% cytosolic transcripts	95.2	74.3	86.2	80.8	79.6	80.3	80.6	76.2	57.6	70.9
# nuclear transcripts	252	3,857	1,845	2,676	3,483	1,741	2,305	4,317	3,256	5,430
% nuclear transcripts	0.9	10.4	7.7	10.4	12.6	8.7	9.0	15.4	14.3	18.0
# genes w/ > 1 expressed transcript	6,737	8,802	6,286	6,626	7,006	5,108	6,579	7,135	6,164	7,547
# genes w/ transcripts of different localization	859	4,309	1,861	2,395	2,893	1,845	2,666	3,260	2,850	3,631
% genes w/ transcripts of different localization	12.8	49.0	29.6	36.1	41.3	36.1	40.5	45.7	46.2	48.1
# genes w/ transcripts of the same biotype and different localization	97	1,164	460	514	564	773	516	727	1,458	900
% genes w/ transcripts of the same biotype and different localization	1.4	13.2	7.3	7.8	8.1	15.1	7.8	10.2	23.7	11.9

Table S5. Gene and transcript localization summary. This table presents the distribution of genes and transcripts identified across the different cell lines analyzed. We report the number of genes and transcripts expressed in each cell line and a summary of their localization. For transcripts, we report the number and the percentage of them with nuclear (localization index $(LI) < 0.4$), or cytosolic ($LI > 0.6$) localization. For genes, we indicate the number and the percentage of them that have expressed transcripts with diverse localization. These were calculated for all genes with multiple transcripts expressed, as well as for genes with multiple transcripts expressed falling within the same annotated biotype category.

References

[1] Xiaomin Dai, Yangmengjie Li, Weizhen Liu, Xiuqi Pan, Chenyue Guo, Xiaojing Zhao, Jingwen Lv, Haixin Lei, and Liye Zhang. Application of rna subcellular fraction estimation method to explore rna localization regulation. *G3 Genes|Genomes|Genetics*, 12(1), November 2021. ISSN 2160-1836. doi: 10.1093/g3journal/jkab371. URL http://dx.doi.org/10.1093/g3journal/jkab371.
